# Prevalent migraine as a predictor of incident hypertension

**DOI:** 10.1093/eurpub/ckab219

**Published:** 2022-01-26

**Authors:** Anitta H Entonen, Sakari B Suominen, Lauri H Sillanmäki, Päivi T Rautava, Katariina Kauniskangas, Pekka T Mäntyselkä, Markku Sumanen, Markku J Koskenvuo

**Affiliations:** 1 Department of Public Health, Faculty of Medicine, University of Turku, Turku, Finland; 2 School of Health Sciences, University of Skövde, Sweden; 3 Research Services, Turku University Hospital, Turku, Finland; 4 Turku University Hospital, Turku, Finland; 5 Department of Public Health, Faculty of Medicine, University of Helsinki, Helsinki, Finland; 6 Healthcare Services, City of Turku, Section of Welfare, Turku, Finland; 7 Institute of Public Health and Clinical Nutrition, University of Eastern Finland and Primary Health Care Unit, Kuopio University Hospital, Kuopio, Finland; 8 Faculty of Medicine and Health Technology, University of Tampere, Tampere, Finland

## Abstract

**Background:**

Migraine has been associated with several diseases. This population-based prospective Finnish postal survey Health and Social Support Study explored whether self-reported migraine predicted incident hypertension independently in a working-age population by utilizing two data sources: the baseline survey from the year 1998 in combination with the follow-up survey data from the years 2003 and 2012 with linkage to the national Social Insurance Institution registry data of the special reimbursement medication for hypertension from 1999 to 2013. The survey follow-up reached until the second follow-up in the year 2012. The register follow-up also included the year 2013.

**Methods:**

The present population-based prospective cohort study, utilizing two different data sources, included 8593 respondents (22.7% response rate) who participated in 1998, 2003, and 2012 but who did not report hypertension at the baseline in 1998, and whose responses could be linked with the Social Insurance Institution registry data from the beginning of 1999 to the end of 2013. The multivariable logistic regression analysis was based on the combined two data sets.

**Results:**

A significant association of self-reported migraine and incident hypertension (odds ratio 1.37; 95% confidence interval 1.20–1.57) prevailed in the multiple logistic regression analysis adjusted for central socio-demographic and health behaviour variables.

**Conclusion:**

Extra attention should be paid to prevention and control of hypertension in working-age migraine patients.

## Introduction

Hypertension is a global health challenge due to its high prevalence and its association with increased morbidity and mortality from cardiovascular diseases.^1^ *Estimated global age-standardized prevalence of hypertension in adults in 2010 was 31.1%, however, with considerable variation between high-income 28.5% (27.3–29.7%), and low- and middle-income 31.5% countries (30.2–32.9%).^1^ Each 20/10 mmHg increase of blood pressure (BP) doubles the risk for cardiovascular disease (CVD).^2^ Although any substantial reduction of BP dramatically reduces the risk for CVD, prevalent hypertension remains a principally uncontrolled public health concern.^2^^,^^3^ Around 386000 persons in Finland had the right to the special reimbursement medication for hypertension granted by the Social Insurance Institution of Finland (Kela) at the end of 2020.^4^ Approximately 2 million of all Finnish adults have elevated blood pressure and one million of them are prescribed with BP-lowering medication.^3^ In spite of this, only 40% of them will reach the treatment goals for hypertension^3^ underlining the significance of the disease as a notable public health concern.

Migraine has for long been considered a disorder without long-term consequences to the brain.^5^ Decades of research has provided evidence for migraine being associated with a potential to progress into a chronic form, and in some patients, to a progressive disorder leading to significant humanistic and societal implications.^6^ Migraine is considered chronic by the Finnish current care summary if there are 15 headache days or more in a month and if the criteria for migraine headache are met in 8 days in a month.^7^ Migraine is ranked among the most disabling diseases for the degree of disability it causes.^8^

Vascular aspects have also contributed to an interest in a possible link between migraine and hypertension but the epidemiological evidence is controversial.^9^ Many studies support the hypothesis that migraine patients have an increased risk of developing hypertension.^10–13^ The focus of the present follow-up study was to explore whether the association of migraine and hypertension adjusted for gender, age, initial measures of occupational training, living alone (rough measure of marital and social support), metabolic equivalent of task (MET), body mass index (BMI), alcohol consumption, and smoking status found in 2003 in our previous study^14^ prevailed during 1998–2013 in the Finnish working-age population.

## Methods

The present study is part of the population-based cohort study, Health and Social Support (HeSSup) Study, which consists of a prospective postal survey and a register-based follow-up. The Population Register Centre granted permission for the use of the study population in 1998 and the initial survey was carried out from September 1998 to February 1999 and was completed with one reminder. The respondents were asked for consent for later linkage of the questionnaire data with selected medical registries [Care Register for Health Care, Register of special reimbursement medications for chronic or severe conditions, the Social Insurance Institution of Finland (Kela) registry, Cancer register, Pension register]. The present study was based on the epidemiological questionnaire data and the Social Insurance Institution in Finland (Kela) registry data, and no invasive procedures were carried out. Thus, the study was scrutinized and approved by the Ethics Committee of Turku University Hospital in 1998.

### Data collection

The original HeSSup study population consisted of a random sample of 64 797 individuals from the Finnish Population Register Centre, stratified according to gender and four age groups: 20–24 (*N* = 16 190), 30–34 (*N* = 16 250), 40–44 (*N* = 16 277) and 50–54 years of age (*N* = 16 080) in 1998. A total of 25898 respondents (59% women) returned the baseline questionnaire in 1998 (40% response rate). According to a non-response analysis of the study, the data were found to be representative of the Finnish working-age population. The follow-up survey in 2003 yielded 19629 acceptable responses (80.2% response rate).^15^

The association of self-reported migraine and a physician confirmed hypertension at the 15 years of follow-up was examined by utilizing two data sources: the questionnaire data of the baseline HeSSup Study in the year 1998 in combination with the follow-up survey data from the years 2003 and 2012 with linkage to the Kela registry data of the special reimbursement medication for hypertension from 1999 to 2013. The youngest age group was excluded due to the extremely low prevalence of hypertension. Thus, the present study included 8593 respondents (22.7% response rate) who participated in 1998, 2003, and 2012 but who did not report hypertension at the baseline in 1998 and whose responses could be linked with the Kela registry data from the beginning of 1999 to the end of 2013. According to two previous non-response analyses of the HeSSup study data by Korkeila et al.^15^ and Suominen et al.^16^ no substantial selection of participants could be observed having taken place, and additionally, the topics dealing with the external validity of the study have been carefully addressed in the HeSSup study.

### Assessment of hypertension and migraine

In the present study, the primary outcome variable, that is, incident hypertension comprised new cases of hypertension indicated by self-report in the survey data of the HeSSup Study and by the rights for the special reimbursement medication for hypertension in the reimbursement category from the Kela register data. With the use of two data sets, untreated or unreported hypertension was eliminated from the study population. Respondents were asked to report their doctor confirmed medical conditions, among these, hypertension. By combining the information with self-reported medication for the last six months for elevated blood pressure or hypertension during the last 12 months, hypertension was also assessed by the rights of anti-hypertensive medication of the special reimbursement category in 2013 covering the total Finnish population. In cases when both conditions were met, hypertension was considered being prevalent. Selected conditions, such as hypertension, entitle an insured person to a special reimbursement of the medication when specified criteria for the Kela reimbursements have been met.^4^ False positive cases in the drug reimbursement registers are very unlikely as the Kela grants financial benefits in relation to chronic diseases and conditions, and medical treatment only after a strict evaluation procedure.^17^ Details of these criteria have been explained in our earlier study.^14^ Subjects with Kela-assessed hypertension at the beginning of the year 1999 were excluded from the study. For simplicity reasons, from here on the variable is called hypertension.

Self-reported migraine was classified according to responses to the initial survey questionnaire in 1998: ‘Has a doctor ever said that you suffer or have suffered from…’ (yes/no). The respondent was classified missing if she/he had not responded to any of the questions at that part of the questionnaire. Migraine was a binary independent variable. Migraine is assessed according to the International Classification of Headache Disorders (ICHD-3, 2013)^18^ criteria. Migraine is classified as migraine with aura (MA) and migraine without aura (MO).^7^ Migraine without aura is considered a more important form of migraine than migraine with aura because the scientific evidence of the effect of different treatment procedures is limited mostly on migraine without aura.^7^

### Other measurements

In order to reduce the influence of confounding factors, adjustments were made for gender, age, occupational training, living alone, MET, BMI, alcohol consumption, and smoking status. Occupational training was classified into three categories: trade/vocational school or less (less than 9 years of education), institutional level (9–12 years) and university level (more than 12 years of education, the reference class). Living alone was used as a rough measure of marital status and social support. MET represented the intensity of physical activity. It was derived from strenuousness × duration of physical exercise and calculated as daily energy expenditure in metabolic equivalent task hours (MET-hours). The strenuousness of weekly physical activity was self-classified (options: physical activity corresponding walking/brisk walking/light jogging/running) together with the mean duration of exercise in each class of strenuousness (options: not at all/<1/2 h/about 1 h/2–3 h/≥4 h/week). MET was the only continuous variable used in the analyses. BMI calculated as weight in kilograms divided by the square of height in metres, was classified as <25 kg/m^2^ or ≥25 kg/m^2^, latter indicating the cut-point of overweight. Alcohol consumption was inquired as to the participants’ habitual frequency and amount of consumed beer, wine, and spirits. The responses were converted into weekly consumption of pure alcohol and classified as follows: low (0–21.99 g/week), moderate (women 22–174.99, men 22–262.99 g/week) and heavy alcohol consumption (women ≥ 175, men ≥ 263 g/week). Smoking status was classified into four categories: not smoking, previous smoker, current regular smoker (less than five daily cigarettes), current regular smoker (five or more daily cigarettes).^15^^,^^19–21^

### Statistical methods

The associations between baseline migraine and hypertension in 2013 were analyzed by the multivariable logistic regression analysis. One or two independent variables at a time were added to the model. Five models were constructed: Model 1: adjustment for gender, Model 2: as model 1 + adjustment for age, Model 3: as model 2 + adjustment for occupational training and living alone, Model 4: as model 3 + adjustment for MET and BMI, Model 5: as model 4 + adjustment for alcohol consumption and Model 6: as model 5 + adjustment for smoking status. The results were reported as odds ratios (OR) and their 95% confidence intervals (CI). The effect modification of age and gender with the prevalence of migraine in relation to hypertension in 2013 was explored by adding an interaction term to the model. *P* values <0.05 were considered indicating statistical significance. All the tests were two-tailed. The SAS System for Windows 9.4/2016 was used for statistical computing.

## Results

Hypertension at follow-up was significantly (*P* < 0.001) more prevalent among the respondents with migraine 465 (26.9%) than among the other subjects 1483 (21.7%), among women as compared to men, among elder people as compared to younger ones, among persons with the least occupational training as compared to those having the highest occupational training, among persons living alone and persons with BMI 25 or higher, with heavy alcohol consumption, and among current regular smokers with five or more daily cigarettes, though there were no big differences between smoking categories (table 1).

**Table 1:** Prevalence of hypertension by 2013 related to the self-reported baseline variables in 1998 (respondents free from hypertension at baseline 1998). Summary of single predictor logistic regression analysis

	Hypertension by 2013^a^	
	No	Yes
Baseline variable	*N* (%)	*N* (%)
Migraine		
No	5363 (78.3)	1483 (21.7)
Yes	1263 (73.1)	465 (26.9)
Gender		
Women	4163 (78.3)	1152 (21.7)
Men	2476 (75.5)	802 (24.5)
Age		
30–34 years	2148 (89.1)	264 (10.9)
40–44 years	2342 (77.6)	677 (22.4)
50–54 years	2149 (68.0)	1013 (32.0)
Occupational training		
<9 years	2833 (74.6)	964 (25.4)
9–12 years	2465 (77.4)	719 (22.6)
>12 years	1305 (83.6)	257 (16.4)
Living alone		
No	4839 (77.8)	1379 (22.2)
Yes	1800 (75.8)	575 (24.2)
BMI		
<25	4141 (85.1)	728 (14.9)
≥25	2475 (67.0)	1221 (33.0)
Alcohol consumption		
Low	2075 (78.2)	577 (21.8)
Moderate	4274 (77.4)	1250 (22.6)
Heavy	279 (68.9)	126 (31.1)
MET (mean)	35.1	31.1
Smoking status		
Not smoking	3033 (79.4)	788 (20.6)
Former smoker	1811 (75.5)	587 (24.5)
Current regular smoker: < 5 daily cigarettes	173 (80.8)	41 (19.2)
Current regular smoker: ≥ 5 daily cigarettes	1119 (73.8)	397 (26.2)

a A reportedly elevated blood pressure or hypertension and in addition medication for elevated blood pressure or hypertension for over six months during the last 12 months or the medication of the special reimbursement rate for hypertension of the Kela registry data derived from the combined HeSSup study subjective data and data from the Kela registry data.

BMI, body mass index; MET, metabolic equivalent of task.

After adjustments for gender, age, occupational training, living alone, MET, BMI, alcohol consumption and smoking status self-reported migraine remained a significant independent predictor for hypertension (OR 1.37; 95% CI 1.20–1.57) in the multivariable logistic regression analysis (table 2).

**Table 2:** Summary of logistic regression analysis results of self-reported migraine at baseline, in 1998 as a predictor of hypertension at follow-up by 2013 (respondents free from hypertension at baseline)

HeSSup Study data and Kela registry data combined^a^	OR (95% CI)
Model 1: adjustment for gender	1.40 (1.23–1.58)
Model 2: as Model 1 + age	1.36 (1.19–1.54)
Model 3: as Model 2 + occupational training and living alone	1.35 (1.19–1.54)
Model 4: as Model 3 + MET and BMI	1.34 (1.18–1.53)
Model 5: as Model 4 + alcohol consumption	1.35 (1.19–1.54)
Model 6: as Model 5 + smoking	1.37 (1.20–1.57)

*Note*: No migraine at baseline = reference value 1.00. *P* < 0.001 for all of the models 1–6 (*P*-values = 0.05 was used as a limit of statistical significance).

a Data derived from the HeSSup study subjective data and the Kela registry data combined.

## Discussion

The aim of the present study was to explore whether self-reported migraine at baseline in 1998 predicted hypertension in a Finnish working-age population during 15 years of follow-up. After adjustments for several potentially confounding variables, individuals with migraine at baseline had an approximately 1.35-fold significantly increased risk of hypertension as compared to individuals without initial migraine. Migraine showed a very similar association explored separately by the HeSSup survey data and the Kela registry-based data for the reimbursement medication for hypertension in our former study^14^ and therefore these two data sources were combined in the present study.

Migraine physiology is not completely understood nevertheless many tools have been used, among them highly advanced imaging techniques, to explore migraine pathophysiology.^6^^,^^9^^,^^22–24^ Most likely migraine headache depends on activation of the trigeminally mediated vascular pathway and dysfunction of central nervous system.^6^^,^^9^^,^^24^ Many drugs used for the prophylactic purposes of migraine attack have been detected by chance and were originally accepted for other medical conditions. It is hypothesized that the renin–angiotensin system, involved in hypertension and in the central nervous system (CNS), could at least partially be responsible for the association between migraine and hypertension^9^^,^^22^ This in turn, might be the explanation to the observations that some angiotensin-converting enzyme (ACE) inhibitors, and angiotensin receptor blockers (ARB) are shown to be effective in preventing migraine attacks as well as beta-blockers suppressing cortical excitability (cortical spreading depression).^22–25^ Recently three new humanized monoclonal antibodies, erenumab among others targeting calcitonin gene-related peptide (CGRP), are indicated for preventative treatment of episodic and chronic migraine in adults.^26^ Part of the migraine medicines is now reimbursed in Finland by the Kela, which enables population-based register research on reimbursed migraine medication in working-age population.^27^

As headache may also be a symptom of hypertension, we tested whether hypertension predicted migraine among people with no initial migraine, but this was not the case (data not shown).

In the present 15-year follow-up study, self-reported migraine predicted hypertension in a Finnish working-age population. This finding is supported by Cirillo et al.^10^ who found a strong association between severe headache and hypertension (OR 1.82; 95% CI 1.39–2.38 for men, OR 1.34; 95% CI 1.08–1.68 for women, and OR 1.51; 95% CI 1.28–1.80 for both genders) in a population study of 1343 participants aged 15–64 years. In turn, in a case–control study of 6102 persons with migraine and 5243 controls by Bigal et al.,^11^ migraine with or without aura was associated with both occurrence of CVD and risk factors for CVD including hypertension. The prevalence of persons with migraine showed a 1.4-fold increased risk of hypertension (OR 1.40; 95% CI 1.30–1.60) as compared to persons without migraine.^11^ Similar association was seen in our previous study.^14^ Rist et al.^12^ have found in their 12-year follow-up study among 29 040 women that women experiencing MA had a 9% [relative risk (RR) 1.09; 95% CI 1.02–1.18] increase in their risk of developing hypertension, while women with MO had 21% (RR 1.21; 95% CI 1.14–1.28) increase in their risk of developing hypertension. Women with previous history of migraine had 15% (RR 1.15; 95% CI 1.07–1.23) increase in their risk of developing hypertension as compared to women with no history of migraine. In a post-hoc analysis, they observed that women who experience MO had a higher risk of incident hypertension (RR 1.10; 95% CI 1.01–1.20) than women with MA.^12^ In a population-based cross-sectional study of 5755 male and female participants aged 20–65 years by Scher et al.,^13^ persons with migraine, especially with MA, had higher CVD risk profile including hypertension than individuals without migraine with women showing stronger risk elevation than men.

The focus of the present study was to explore whether self-reported migraine predicted hypertension among randomly selected Finnish working-age persons during a 15-year follow-up.

### Methodological aspects

In the present study, the analyses were adjusted for the potential risk factors of hypertension. Since the prevalence and incidence of hypertension are increasing by age, interaction terms comprising gender and age and the prevalence of migraine in relation to incident hypertension in 2013 were included as part of the logistic regression analysis. Both interaction terms were statistically insignificant (gender × migraine = 0.427 and age × migraine = 0.209). Due to this, final analyses were performed for women and men combined. The youngest age group (20–24 years) was excluded because the prevalence (*N* = 28/2.63%) of hypertension was extremely low among them. The procedure could be regarded as rather strengthening than weakening the validity of the study results. The form of migraine, migraine with or without aura symptoms, cannot be inferred from the study data because the presence of aura symptoms was not separately inquired.

Migraine was classified according to self-reported data. Self-report again could be expected rather weaken than strengthen the associations found since under-reporting is more likely than over-reporting, at least in the milder cases. Self-report is not an ideal measure of a disease, and the authors recognize the problem. Abrignani et al.^28^ found an ICHD-II-based questionnaire to be valid for use in epidemiologic research. In order to validate self-reports of physician-diagnosed migraine, we examined the association of self-reported migraine in the survey years 1998 and 2003 and the recorded use of triptan treatment for migraine by the Kela registry data between 1998 and 2003. Out of 664 respondents who had used triptans according to the Kela registry, 458 (68%) reported having migraine (OR 10.16; 95% CI 8.55–12.09). We also tested the effects of differences in number of BP measurements on the incidence of hypertension between those with migraine and those without migraine. Proportion of measurements within a year was a bit higher in the migraine group as compared to the non-migraine group (55.4 vs. 61.2%) but mean values did not differ statistically significantly (Cochran–Mantel–Haenszel Mean scores difference test, *P* = 0.3558).

The study was a longitudinal one with a representative sample of corresponding age from the Finnish population. The cohort was large enough for studying the aetiological risk factors and changes in health states. The strength was the use of reliable Kela registry data in confirming self-reported hypertension. The results of the present study could be considered consistent and extrapolated as compared to former studies. The present 15-year follow-up time produced profound results for gradually progressing hypertension. Finally, it must be mentioned that the registry-based follow-up extended 1 year longer than the survey follow-up. Hence, some cases of self-reported hypertension could have been missed due to time lag before individuals are granted their right to subsidized medication. However, this has potentially a weakening rather than a biased strengthening effect on the principal results.

## Conclusion

Self-reported migraine at baseline was associated with an increased risk of hypertension in the Finnish working-age population in a 15-year follow-up. Migraine pathophysiology is on its way to be clarified thus deepening the understanding of the associations between migraine and hypertension. Also, the treatment of the migraine patients might be intensified. However, the background of the association studied is multifactorial and further studies are needed. Attention should be paid to both prevention of hypertension and its early detection in working-age migraine patients. This emphasizes the importance of regular blood pressure monitoring at all levels of health care.

## Funding

The HeSSup Study questionnaires in 1998, 2003, and 2012 were supported by the Finnish Academy [projects: 41290, 77560 and 105195].

*Conflicts of interest*: None declared.

Key points

- Both migraine and hypertension are highly prevalent among the Finnish working-age population.
- The public health and individual significance of migraine may still not be fully recognized.
- This follow-up study found an increased risk of hypertension among those with previously diagnosed migraine.
- The increased risk of hypertension should imply greater attention when examining patients with migraine.

## Data availability

The data underlying this article cannot be shared publicly for the privacy of the individuals that participated in this study.
